# Critical zone agrohydrology: An integrative paradigm for agricultural water sustainability

**DOI:** 10.1093/pnasnexus/pgag203

**Published:** 2026-06-05

**Authors:** Ying Zhao, Li Guo, Jian Liu, Jie Xue, Jinzhao Liu, Yingkai Chen, Xinyan Cai, Kenneth C Carroll, Esteban Jobbagy, Steven P Loheide, Kathleen M B Boomer, Yanping Li

**Affiliations:** Dongying Base of Integration between Industry and Education for High-quality Development of Modern Agriculture, Ludong University, Dongying 257509, China; Global Institute for Water Security, University of Saskatchewan, Saskatoon, SK, Canada S7N 3H5; State Key Laboratory of Hydraulics and Mountain River Engineering, College of Water Resource and Hydropower, Sichuan University, Chengdu 610065, China; Norwegian Institute of Bioeconomy Research (NIBIO), Ås 1431, Norway; Xinjiang Institute of Ecology and Geography, Chinese Academy of Sciences, Urumqi 830011, China; State Key Laboratory of Loess and Quaternary Geology, Center for Excellence in Quaternary Science and Global Change, Institute of Earth Environment, Chinese Academy of Sciences, Xi’an 710061, China; Shandong Academy of Agricultural Sciences, Jinan 250100, China; Shandong Academy of Agricultural Sciences, Jinan 250100, China; Department of Plant and Environmental Science, New Mexico State University, PO Box 30003, MSC 3Q, Las Cruces, NM 88003-8003, USA; Grupo de Estudios Ambientales—IMASL, CONICET, Universidad Nacional de San Luis, San Luis, HHW D5700, Argentina; Department of Civil and Environmental Engineering, University of Wisconsin—Madison, WI 53706, USA; Foundation for Food and Agriculture Research, 401, 9th Street NW, Suite 730, Washington, DC 20004, USA; Global Institute for Water Security, University of Saskatchewan, Saskatoon, SK, Canada S7N 3H5

## Abstract

Meeting rising food demand under intensifying climate variability, soil degradation, and groundwater decline requires agriculture to produce more with less freshwater. We advance critical zone agrohydrology (CZA) as a unifying framework that treats agricultural landscapes as human-managed critical zones—coupled systems extending from canopy to bedrock and operating from seasons to decades. CZA is organized around the four deeps (deep time, deep depth, deep coupling, and deep practice) and operationalized through a 5M cycle of measuring, mapping, monitoring, modeling, and managing. This perspective expands conventional agrohydrology by accounting for long-term soil change, subsurface storage and flow, biogeochemical feedbacks, and human decision-making, thereby linking field efficiency with basin sufficiency. We illustrate implications for multifunctional soil management, nutrient-loss control, salinity rehabilitation, drought resilience, managed aquifer recharge, and cross-scale governance. By reframing agriculture as a potential contributor to aquifer stability, water quality, carbon storage, biodiversity, and durable productivity, CZA offers a practical pathway toward more resilient and basin-aware agricultural water management.

## Introduction

**Figure 1:**
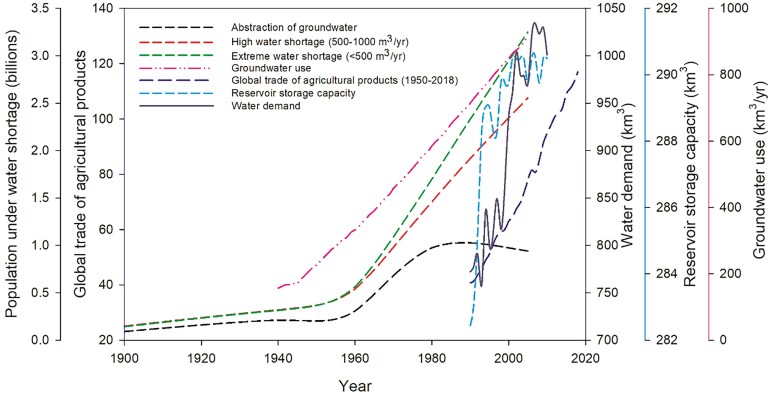
Long-term changes in agricultural water use and water stress under population growth, land-use change, and climate variability. Synthesized from Kummu et al. (1), Siebert et al. (2), Famiglietti (3), Schwarz et al. (4), and Di Baldassarre et al. (5).

Global food and water security are under mounting pressure. Agricultural expansion and intensification have sharply increased freshwater use, while climate variability, soil degradation, and groundwater decline are narrowing the room for error (Fig. 1). Consumptive water use rose rapidly during the twentieth century, and severe water scarcity now affects billions of people for at least part of the year (6–8). In many arid and semiarid regions, intensive irrigation has overdrawn rivers and aquifers, producing groundwater decline, soil desiccation, and land subsidence (2, 9). Agriculture therefore must produce more food with less water while sustaining the ecosystems that support long-term productivity.

**Figure 2:**
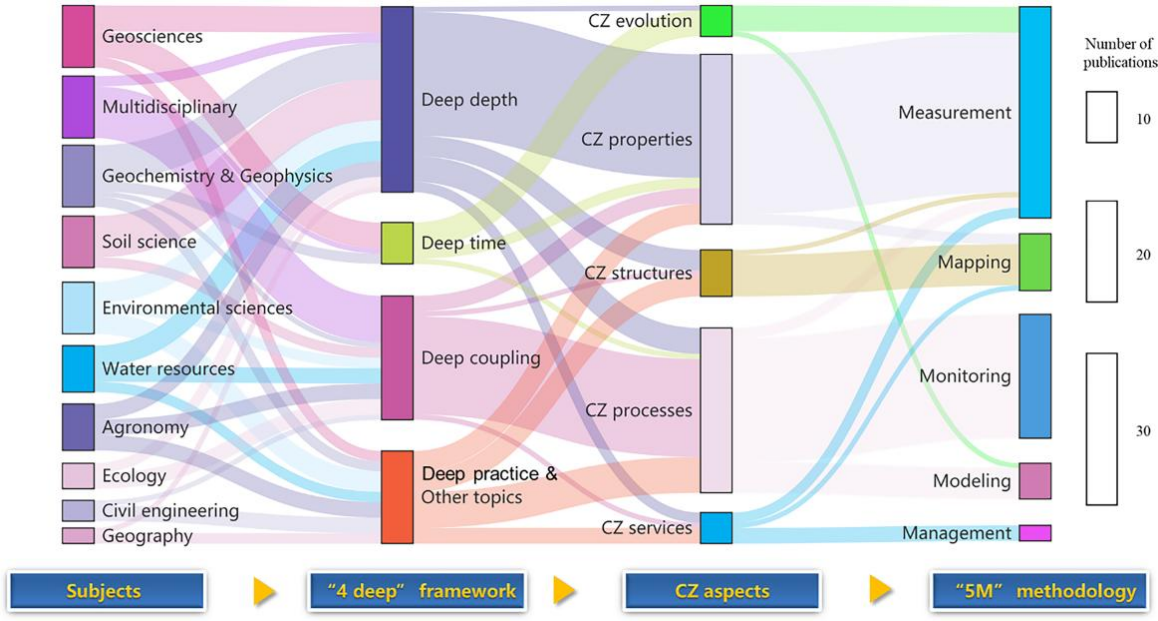
Disciplinary context of CZA across adjacent hydrologic and agroecosystem sciences.

**Figure 3:**
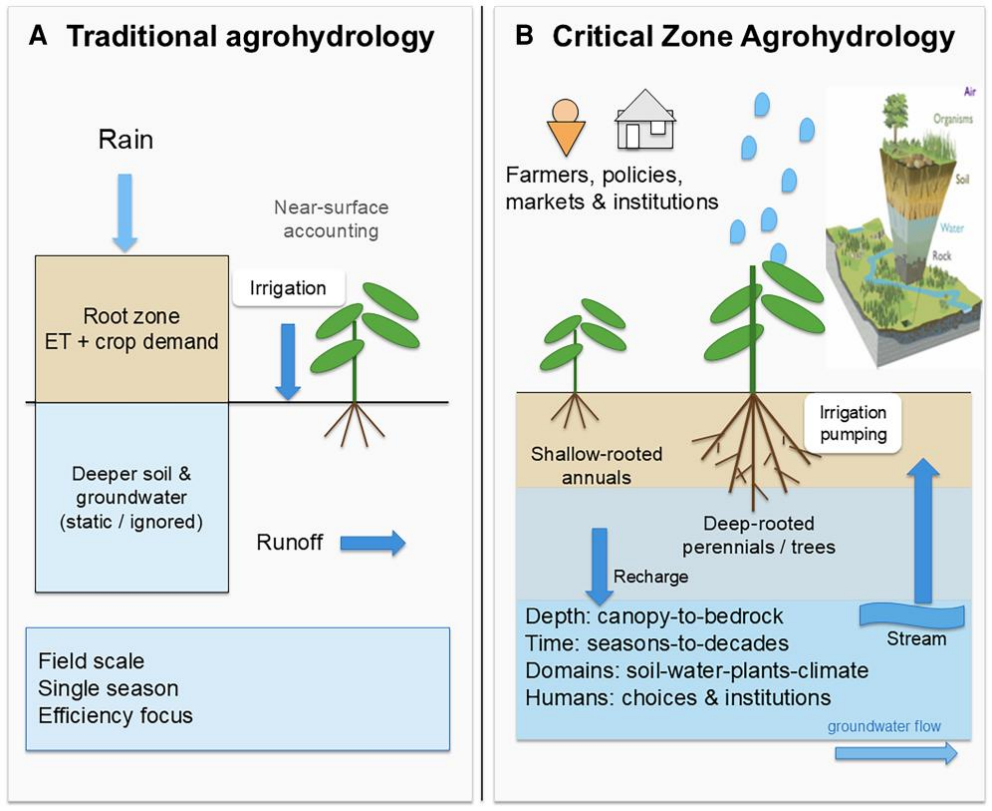
From traditional agrohydrology to CZA. A) The classical field-scale, single-season focus on irrigation scheduling and water-use efficiency. B) The full CZ—from canopy to bedrock and from field to catchment—linking soil, groundwater, surface water, climate, vegetation, and human decisions.

Agrohydrology emerged to bridge agronomy and hydrology in pursuit of more efficient water use (Figs. 2 and 3). Its core emphasis has been field- to farm-scale water balances, irrigation practices, and short-term crop responses. Advances in irrigation management, deficit irrigation, mulching, conservation tillage, crop simulation, and remote sensing have improved water productivity and expanded on-farm decision tools (9–15).

Yet conventional agrohydrology often remains surface-oriented and short-term. By focusing mainly on the plow layer and root zone, it can miss slow changes in soil structure, organic matter, salinity, groundwater storage, and subsurface flow paths. Practices that optimize short-term yield may therefore degrade long-term resilience by depleting aquifers, amplifying drought vulnerability, or mobilizing salts (16–18).

To address these gaps, we introduce critical zone agrohydrology (CZA), an integrative perspective rooted in critical zone (CZ) science—the study of Earth's near-surface “living skin,” where rock, soil, water, air, and life interact (19, 20). Viewing farmland as a human-modified CZ frames agriculture as a coupled biological, geological, hydrological, and managed system. CZA does not assume that deep horizons are always measured; it requires that they be considered conceptually and, where feasible, measured or inferred.

This perspective outlines the conceptual foundations of CZA, presents the 5M cycle (measuring, mapping, monitoring, modeling, and managing) as an operational roadmap, and applies the framework to major agricultural water challenges.

## Agrohydrology and CZ science: strengths and limitations

### Agrohydrology's strengths and blind spots

#### Strengths of traditional agrohydrology

Agrohydrology has traditionally emphasized water management at the field and farm scales—tracking rainfall, irrigation, runoff, soil moisture, and crop water use. This focus has driven major advances in agricultural water efficiency and productivity. Improved scheduling techniques, modern irrigation technologies (e.g. drip and sprinkler systems), and better drainage methods allow farmers to match water supply with crop demand more precisely. Crucially, agrohydrology is an applied discipline oriented toward solving immediate farm-level challenges. It accounts for crop requirements, farmer practices, and economic constraints, and its engineering mindset has yielded practical tools (such as irrigation scheduling guidelines and field-level water budgets) that are readily adopted by farmers. In practice, traditional agrohydrology is especially strong in addressing near-term, field-scale water-management challenges: optimizing water use for crop production under current socioeconomic conditions.

#### Blind spots and limitations

Despite these achievements, conventional agrohydrology often treats farms as isolated units, overlooking basin-scale linkages and long-term dynamics. Efficiency gains can reduce on-farm losses yet also diminish recharge and downstream flows, and “saved” water is often reinvested to expand irrigation, triggering rebound effects (7, 21). Its short, surface-oriented lens also treats groundwater as a static black box, missing slow recharge, deep vadose zone and bedrock processes, and long-term soil degradation. Human behavior is likewise too often externalized, even though crop choice, pumping, technology adoption, and policy co-govern water outcomes.

These blind spots have tangible consequences worldwide. In major irrigation regions, decades of intensive pumping have lowered the water tables and weakened connections between river and aquifers (3, 18). In dryland basins, replacing deep-rooted native vegetation with shallower annual crops can raise water tables and mobilize salts, contributing to secondary salinization (22). Neither outcome is well captured by short-term, field-scale approaches focused mainly on immediate yield. Moving forward, agrohydrology must retain its pragmatic, solution-oriented ethos while broadening its scope: deeper soils, longer timescales, larger spatial scales, and the human dimensions of decision-making beyond the farm gate. Only by integrating biophysical and socioeconomic systems can field-level gains translate into basin-scale resilience.

### CZ science's insights and limitations

#### Insights from CZ science

CZ science provides an integrative, multiscale lens that helps address key gaps in agrohydrology (Fig. 2). It treats Earth's near-surface—from canopy and soils through weathered bedrock to groundwater—as a single, coupled system, enabling fuller accounting of causes, effects, and trade-offs. This whole-system perspective links soil, water, biota, atmosphere, and human management, revealing feedbacks that shape water availability, quality, and crop performance. For example, studies of weathered bedrock and rock moisture show that subsurface storage can sustain plant water use during severe drought, while work on dynamic soil structure shows how biological activity and management alter hydraulic function (23–25). The vertical or “deep” dimension extends analysis far below the plow layer, highlighting hidden controls such as rock-stored moisture, macropore and fracture flows, and soil–bedrock interactions that regulate infiltration, root access, recharge, and salinization. A deep-time perspective spans seasons to millennia, situating present conditions within geological history and land-use legacies. It encourages management that builds slow variables—soil carbon, structure, and groundwater storage—critical for long-term resilience. By integrating biogeohydrological processes with human decisions across depths and timescales, CZ science supports agricultural strategies that are predictive, robust, and scalable.

In sum, CZ science is holistic, vertically deep, temporally deep, and human inclusive. It brings into view downstream connections, hidden subsurface processes, and long-term trajectories while explicitly incorporating people as agents within the system. In agricultural settings, this means that fields receiving similar rainfall or irrigation may respond differently because of subsoil architecture, fracture networks, soil-biological structure, or legacy management, not merely because of surface inputs (25–27). These insights show that sustainable agricultural water management must address the whole system, not just the field.

#### Limitations of CZ science (for agriculture)

Despite its strengths, CZ science has limitations when applied to farming. Early research emphasized natural ecosystems and system diagnosis more than near-term management design, so insights can remain detached from farm or water-agency decisions. Socioeconomic dimensions are also underrepresented: many CZ models still treat people as external drivers rather than coupled actors, seldom engaging farmer incentives, governance, or policy. This limitation has been recognized more broadly in recent CZ investigation, which calls for extending both the spatial reach of CZ science and its explicit treatment of human impacts and human-modified systems (28). Practical uptake is further hindered by data- and expertise-intensive methods. Deep subsurface characterization and long-term monitoring are scientifically valuable, but they must be translated into simpler indicators and decision-support tools if they are to guide agricultural practice. Examples include converting geophysical imaging, isotope tracing, and long-term observatory records into farm-scale indicators, and incorporating farmer learning preferences, local knowledge networks, and incentives into human-modified CZ analysis (29–31). Scaling remains difficult as well, because findings from heavily instrumented observatories do not automatically transfer to heterogeneous agricultural regions. These limits are precisely why an explicitly integrative CZA framework is needed.

Despite these challenges, combining CZ science with agrohydrology offers a promising path forward: agrohydrology contributes practical, human-centered solutions, while CZ science adds holistic depth across space and time. CZA uses this complementarity to translate CZ examples and evidence into agricultural diagnosis and management.

## Proposal of CZA

### Why agriculture needs a CZ framing now

Agrohydrology has been highly effective at improving field-scale water management, but its analytical center of gravity remains with water balances, nutrient transport, and within-season optimization. CZ science, by contrast, emphasizes multiscale flow paths, subsurface architecture, and long-term process interactions. That difference matters because water in agroecosystems is not only a flux; it is also a storage, a transport medium, and a regulator of biogeochemical and ecological change (Fig. 3). A field may appear efficient while depleting groundwater, shifting risk downstream, or masking salinity and subsurface constraints. These are quintessential CZ problems because they arise from vertical heterogeneity, lateral connectivity, and temporal lags that conventional field accounting often cannot resolve.

CZA is therefore proposed not as a replacement for agrohydrology, but as its strategic expansion. It broadens what counts as relevant evidence for explanation and management: from root-zone accounting to canopy-to-bedrock diagnosis, from within-season optimization to long-term storage trajectories, and from field performance alone to indicators that remain meaningful at farm, district, and basin scales. In doing so, CZA makes agricultural water problems more diagnosable, comparable across scales, and tractable under climatic and socio-environmental change (29, 32, 33).

### Rationale for developing CZA

#### Addressing blind spots in water accounting

Traditional agrohydrology often resolves field-scale balances but only partly captures return flows, cross-farm reuse, subsurface storage change, and lagged basin responses (32). These omissions matter because field-scale efficiency does not automatically produce basin-scale savings. CZA therefore reconnects field accounting with basin outcomes by combining noninvasive sensors, isotopic tracing, hydrogeophysical surveys, and data-assimilating models that quantify deeper storage, delayed fluxes, and spatial redistribution (29, 33).

#### Incorporating long-term and subsurface processes

Contemporary water management often overlooks multidecadal soil change, groundwater dynamics, and bedrock influences even though they shape sustainability. CZA explicitly includes soil formation and degradation, slow shifts in organic matter and structure, and surface-groundwater interactions. Its practical claim is not that deep resources are universal but that deep processes are context specific and must be evaluated rather than ignored. Long-term monitoring and climate-informed modeling can therefore detect salinization, groundwater decline, or soil degradation early enough to support proactive responses such as soil-carbon restoration or managed aquifer recharge (18, 27).

#### Linking biophysical and human components

Agricultural water outcomes emerge from coupled natural-human systems. Farmer and policy decisions feed back into hydrology, and efficiency technologies can even increase total water use through rebound effects. The added value of CZA is therefore to place water, energy, nutrients, crops, economics, behavior, and policy within the same analytical frame (21, 34, 35). This coupling allows interventions to be judged not only by agronomic performance but also by adoption constraints, governance context, and distributional consequences.

#### Translating science into practice

A central aim of CZA is to turn complex system understanding into actionable guidance. Deep practice operationalizes the three traditional deeps (deep time, deep depth, and deep coupling) (20) through co-production, clear metrics, and iterative learning (36, 37). The goal is to develop context-specific playbooks, extension pathways, digital tools, and policies that farmers and water managers can actually use.

Although CZ approaches are well established in natural ecosystems, they remain underused in agroecosystems. Bringing agrohydrology into the CZ lens makes it possible to trace anthropogenic fingerprints in the water cycle and move from prediction alone toward decision intelligence that links pattern recognition with intervention, accountability, and basin outcomes (21, 38).

### Interplay with related hydrologic disciplines

**Figure 4:**
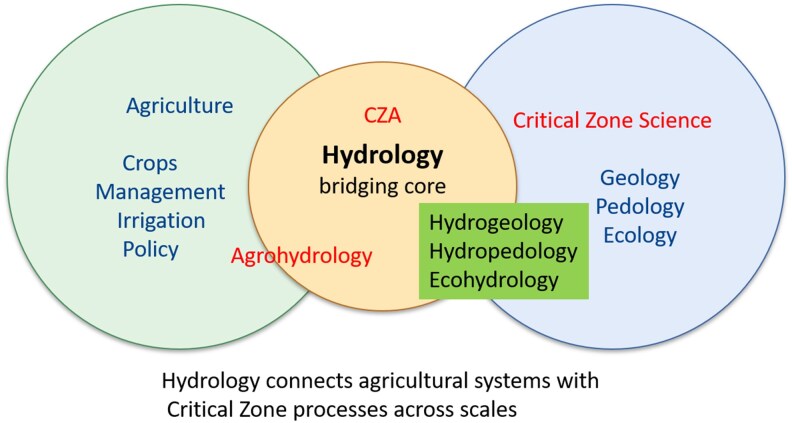
Positioning of CZA relative to agriculture, hydrology, and related disciplines.

Agrohydrology sits among several hydrologic subdisciplines that each illuminate part of the agricultural water system (Fig. 4). Hydrogeology focuses on groundwater, hydropedology on soil-landscape controls, ecohydrology on water-biota feedbacks, and hydrometeorology on land-atmosphere exchange. CZA is not a parallel discipline but a synthesis that embeds agrohydrology within a fuller CZ view of how agricultural landscapes function.

This synthesis becomes operational when those disciplines are treated as interacting components of a single decision framework. Hydrogeology brings recharge and aquifer sustainability into view; hydropedology explains how soil structure and topography control infiltration, storage, runoff, and compaction risk; ecohydrology clarifies how roots and vegetation shape uptake and drought buffering; and agrometeorology links forecasts and climate variability to planting, irrigation, drainage, and fertilizer timing (18, 24, 34, 39, 40). Together they convert fragmented process knowledge into an integrated operating picture for diagnosis and management across field, district, and basin scales.

### Conceptual framework: the four “deeps” of CZA

**Figure 5:**
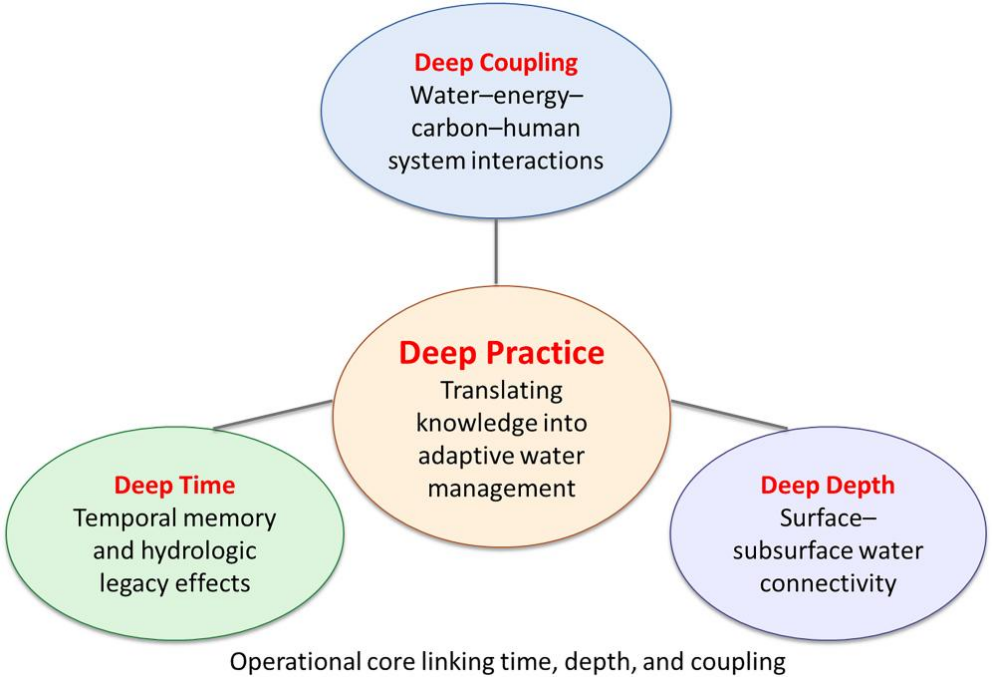
Conceptual framework of CZA organized around the four deeps.

CZA combines agrohydrology's practical focus on water management with CZ science's emphasis on whole-system structure, long-term change, and cross-scale interaction (19, 20). Its central premise is that sustainable agricultural water management requires a deeper view of agroecosystems across time, depth, process, and practice. We organize this perspective around four complementary dimensions—the four deeps: deep time, deep depth, deep coupling, and deep practice (Fig. 5). The first three extend core insights from CZ science, while the fourth renders them operational for agricultural systems (30). Together, they provide a compact logic for diagnosis, intervention, and adaptive management.

“Deep time” emphasizes that agricultural water systems are shaped not only by seasonal fluxes but also by slow variables and long-term trajectories. Multiyear groundwater decline, soil organic carbon change, and salinity evolution condition system responses and recovery potential (16, 18, 41). The working hypothesis is that slow variables regulate fast hydrologic responses such as evapotranspiration, runoff, and drought sensitivity. Management therefore shifts toward trajectory-based stewardship, including rotations, residue retention, reduced tillage, and long-term monitoring of soil and water dynamics.

“Deep depth” extends analysis beyond the plow layer to the soil–regolith–bedrock–aquifer continuum as a hydraulically coupled system. Multidepth soil-moisture observations, groundwater dynamics, and geophysical imaging reveal subsurface controls on storage, recharge, capillary rise, and preferential flow (18, 23, 27). The central hypothesis is that deeper storage buffers drought, while bypass pathways accelerate water and nutrient loss. Management therefore targets subsurface constraints through subsoil amelioration, deep-rooting systems, conjunctive use, and managed aquifer recharge where feasible.

“Deep coupling” recognizes that agricultural water outcomes emerge from feedbacks among hydrology, energy, nutrients, crops, and human decisions. Field-scale efficiency gains do not guarantee basin-scale sustainability if incentives and governance induce rebound effects (21, 34, 35). The role of deep coupling is therefore to integrate biophysical processes with behavior, economics, and policy, ensuring that interventions are evaluated not only for performance but also for system-wide consequences.

“Deep practice” operationalizes the other three dimensions by translating system understanding into repeatable decisions, measurable outcomes, and adaptive learning. It emphasizes actionable indicators, co-produced knowledge, and feedback loops linking monitoring to management (30, 31, 36, 37). The key premise is that transparent metrics and locally grounded playbooks improve adoption and decision quality. Deep practice thus converts conceptual integration into implementable strategies that can be tested and scaled.

Table 1 distills the four deeps into a compact decision framework, linking diagnostic focus, working logic, and management implications. Read together with the paragraph treatment above, it functions as a bridge between the conceptual core of CZA and the implementation agenda developed in the following sections. Taken together, the four deeps move CZA from conceptual synthesis toward an operational framework. Deep time identifies trajectories, deep depth reveals hidden controls, deep coupling explains cross-system feedbacks, and deep practice translates understanding into action. The table therefore serves not only as a summary but also as a compact map linking theory to implementation.

**Table 1:** The four deeps of CZA as an integrated decision framework linking diagnosis, mechanism, and management.

Deep	Core emphasis	What it adds to agrohydrology	Management implication
Deep time	Slow variables, legacies, and trajectories	Connects seasonal water use to decadal soil and groundwater change	Track soil carbon, salinity, and aquifer trends; manage for resilience, not only yield
Deep depth	Surface-to-groundwater continuum	Brings regolith, bedrock, recharge, and capillary rise into diagnosis	Target subsurface constraints, conjunctive use, and managed aquifer recharge where feasible
Deep coupling	Biophysical and human feedbacks	Integrates hydrology with incentives, behavior, and governance	Evaluate interventions for both performance and adoptability
Deep practice	Operationalization and adaptive learning	Turns diagnosis into indicators, actions, and iterative adjustment	Use playbooks, dashboards, and feedback loops to support implementation

## Implementing CZA: the 5M cycle

**Figure 6:**
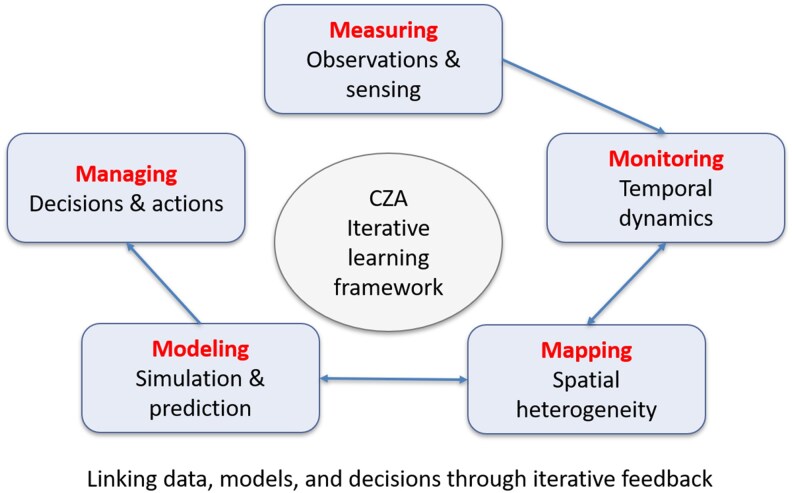
Illustrative components of the 5M cycle in CZA.

Translating the broad concepts of CZA into actionable science and management requires a structured approach. We propose the 5M cycle—measuring, mapping, monitoring, modeling, and managing—as an iterative workflow that links observation, diagnosis, and intervention across the agrohydrologic system (Fig. 6). The 5M cycle functions both as a research methodology and as a decision-support structure, ensuring that data collection informs management and that management outcomes feed back into new measurements. Crucially, the 5M cycle is not a linear checklist but a continuous loop: field data (“measuring”) are transformed into spatial (“mapping”) and temporal (“monitoring”) insights, which feed into predictive tools (“modeling”); model outputs then guide interventions (“managing”), whose effects are measured again, restarting the cycle. This structure enables adaptive learning and can be applied at scales ranging from individual farms to entire watersheds.

Each element of 5M operationalizes the four deeps: long-term monitoring embeds deep time; subsurface-focused measuring and mapping capture deep depth; integrative modeling enables deep coupling of processes (including human factors); and on-ground managing embodies deep practice. The cycle is flexible, multiscaled, and context-specific, adaptable to irrigated districts (tracking water deliveries, efficiency, and aquifer dynamics), rainfed regions (focusing on rainfall capture and drought resilience), high-technology farmlands (leveraging advanced sensors and infrastructure), or saline–alkaline lands (emphasizing mapping and remediation). In all cases, the goal is to link measurements to management in an evidence-driven, feedback-oriented cycle.

### Measuring

Foundational observations span plot to watershed scales: precipitation, soil-moisture profiles, evapotranspiration, runoff, groundwater levels, and indicators of long-term change (e.g. soil organic matter or salinity). A combination of traditional instruments and new technologies provides these data. For example, rain gauges and flow meters measure water inputs and outputs at field and canal scales, while eddy-covariance flux towers and satellites enable field- to regional-scale estimation of evapotranspiration and vegetation water stress (14, 42). Subsurface-focused methods—such as electrical and electromagnetic surveys (electrical resistivity tomography for resistivity and electromagnetic induction for conductivity), cosmic-ray neutron sensing, and even passive gravity measurements—can illuminate deep soil-moisture and groundwater storage changes, revealing what lies beneath the plow layer (43). Isotopic tracers (e.g. using δ^18^O and δ^2^H in water) help partition fluxes and identify source contributions to plant water uptake or recharge (29, 33, 44). Crucially, measurements are chosen and scaled to the context: in water-scarce irrigation districts, flow gauges and pump logs quantify withdrawals and conveyance losses; in dryland farms, nested soil-moisture sensors at multiple depths capture infrequent but critical infiltration events; high-tech commercial farms often deploy dense Internet of Things (IoT) sensor networks for real-time microclimate and soil data (45, 46), and on saline–alkaline land, electromagnetic surveys map soil electrical conductivity to locate salt-affected zones (47, 48). The aim is not data abundance per se, but diagnostic insight—for example, distinguishing evaporation from transpiration, identifying hidden subsurface constraints, and quantifying where water is stored, lost, or made accessible within the agricultural system.

### Mapping

Spatializing observations reveals heterogeneity that is critical for precision management. Digital soil mapping and subsurface surveys can identify key properties like water-holding capacity, restrictive layers (hardpans), salinity hotspots, and depth to groundwater (39, 47). Subsurface mapping with geophysical tools (e.g. EMI and ground-penetrating radar) helps locate buried features, such as clay pans or gravel lenses that influence moisture distribution (49). Remote sensing provides continuous maps of vegetation indices and surface moisture across fields and regions—Normalized Difference Vegetation Index and thermal infrared imagery identify crop vigor and water-stress patterns, while microwave sensors map surface soil moisture (14, 42). Overlay analyses combining multiple maps (e.g. yield vs. soil moisture vs. elevation) can pinpoint persistent wet/dry spots and their causes, guiding site-specific interventions, such as variable-rate irrigation or targeted drainage (50). In practice, modern high-standard farmlands benefit from detailed topographic and soil maps to design laser-leveled fields and efficient irrigation layouts, whereas in extensive arid regions, satellite-derived maps of vegetation and albedo can reveal where runoff concentrates or which areas are most drought-prone. For saline–alkaline terrains, repeat salinity mapping—via EMI or hyperspectral imagery—delineates salt distribution and tracks remediation progress (48). Spatial mapping therefore provides the basis for diagnosing where constraints differ across the landscape and for targeting interventions more precisely than uniform, field-wide management allows.

### Monitoring

Temporal observation programs, ranging from high-frequency sensor streams to seasonal and annual surveys, track trends, thresholds, and system responses to events and management actions. We emphasize, however, that deep soil moisture, groundwater, and flux observations remain sparse in many agricultural regions, so monitoring strategies must be fit for purpose rather than assumed to be uniformly dense. Long-term tracking of groundwater levels, soil moisture and quality, and ecosystem health metrics embeds a deep-time perspective in management and helps detect slow changes such as aquifer decline or soil degradation (16, 18). Monitoring can draw on wireless sensor networks, drone imaging, satellite archives, telemetry in irrigation districts, and simpler community-based systems, depending on context and data availability (42, 45, 46). Its role, however, extends beyond documenting current conditions. Monitoring is also needed to detect thresholds, reveal lagged responses, and distinguish transient variability from directional system change. In practice, this means tracking not only crop stress or irrigation performance but also slower indicators such as groundwater recovery, salinity trends, and soil structural decline. Within CZA, monitoring therefore functions as the temporal bridge between measurement and management, providing the evidence needed to evaluate whether interventions are producing durable benefits rather than short-lived gains.

### Modeling

Models synthesize observations and understanding into predictive tools, enabling exploration of scenarios and serving as “virtual laboratories.” Here, it is important to distinguish illustrative legacy models from the newer coupled approaches that motivate CZA. Older semi-distributed watershed models can still be useful entry points, but they are not sufficient on their own to resolve deeper storage, two-way groundwater coupling, or human decision constraints. The revised emphasis is therefore on coupled crop-groundwater models, data assimilation, agent-based decision modules, and digital-twin-style scenario testing (13, 51–53). In practice, modeling in CZA is used to test, implement, monitor, and recalibrate, thereby reducing uncertainty while identifying thresholds, unintended consequences, and robust options under changing climate and policy conditions (35). Its value lies not only in forecasting but also in integrating observations across depths, compartments, and timescales into a consistent decision framework. Well-constrained models can help distinguish whether similar surface outcomes arise from contrasting subsurface causes—for example, shallow rooting, deep drainage, perched water, or groundwater decline—and can therefore guide more targeted intervention. In this sense, modeling is not a substitute for measurement and monitoring, but the analytical component that links them to scenario evaluation and management design.

### Managing

Management closes the loop, translating knowledge into action while embedding feedback for learning—deep practice in action. Field-level practices include conservation tillage and cover cropping to enhance soil structure and water retention; precision irrigation guided by sensors and forecasts; controlled or subsurface drainage; and agroforestry or buffer strips for water regulation and habitat (11, 12, 36). At larger scales, management can involve building recharge basins or check dams for managed aquifer recharge (MAR) (54, 55), constructing wetlands for nutrient removal (26), and upgrading canals to reduce conveyance losses (26, 56). Policy instruments—such as water allocation limits, tiered pricing, tradable water rights, and payments for ecosystem services—align farm incentives with basin sustainability goals (1, 21, 37). Implementation pathways differ by context: irrigated districts might prioritize delivery scheduling reforms, rotational water supply, and pumping restrictions; arid regions emphasize rainwater harvesting, drought-tolerant crop varieties, and climate-informed planting schedules; high-technology farmlands integrate digital decision support and automation; saline–alkaline lands focus on improved drainage, judicious freshwater leaching, gypsum amendments, and salt-tolerant crop rotations (48). Embedding the 5M approach into agricultural extension services and policy frameworks can broaden its practical impact, for example by linking incentives to performance metrics such as crop per drop, distribution uniformity, soil health, recharge, or reduced pollutant loss. In this sense, managing within CZA means iteratively translating evidence from data, maps, and models into practices and policies that remain responsive to local context and system feedbacks.

### Linking the 5M cycle

In essence, the 5M cycle links observation, interpretation, and intervention within a single adaptive framework. Measuring establishes the observational basis of the system; mapping reveals where constraints and opportunities differ across the landscape; monitoring tracks how those patterns evolve through time; modeling integrates these observations into scenarios and decision support; and managing translates that understanding into action. The strength of the framework lies not in any single component but in the iterative connections among them. Data sharing and common analytic standards remain limited in many agricultural settings, which constrains integrated water management. For this reason, operationalizing the 5M cycle is as much an institutional task as a technical one. One vision gaining traction is the establishment of agricultural CZ observatories on working farms, where surface, subsurface, ecological, and management variables can be tracked together over time (20). Such efforts embody deep practice by linking scientific diagnosis with real-world decision-making and by creating a platform through which farmers, managers, and researchers can evaluate whether interventions improve agricultural water sustainability across seasons and scales.

## Applications of CZA

### Enhancing multifunctionality of agroecosystems

A CZA perspective reframes soil management from a fertility problem to a coupled storage, transport, and resilience problem. Practices that build soil organic matter and stabilize structure—residue retention, reduced tillage, cover crops, and diversified rotations—improve infiltration, root-zone storage, and resistance to both drought and intense rainfall (11, 12, 25, 41). In this view, soil is not only a substrate for crops but also distributed water infrastructure.

A CZ perspective also incorporates riparian buffers, grassed waterways, farm ponds, wetlands, and agroforestry strips as integral parts of the agrohydrological system. These features intercept sediment and nutrients, promote infiltration, store runoff, and moderate microclimate. Although they occupy small areas of annual cropland, their water quality, flood mitigation, habitat, and yield-stabilization benefits can outweigh the foregone acreage (12). CZA provides a framework for assessing such co-benefits, helping justify these designs in economic and policy terms.

The framework is equally useful for diagnosing trade-offs. Practices that maximize yield or field-level efficiency can still pollute groundwater, reduce recharge, or aggravate salinity if leaching and return flows are ignored (57). CZA helps evaluate where nature-based or structural interventions are most likely to alter recharge, runoff, nutrient retention, and drought buffering, and whether those gains hold at basin scale or lead to unintentional consequences.

### Nutrient leaching and water quality

Nutrient losses show why a CZ framing matters. Coupling hydrologic transport with biogeochemical transformation allows managers to identify where nutrients are generated, stored, intercepted, or removed. Strategic monitoring and integrated modeling can therefore guide the placement of wetlands, filter strips, bioreactors, or drainage controls to intercept nitrate before it reaches streams (26, 58).

A CZA-guided strategy aligns hydrologic residence times with biogeochemical opportunity windows for nutrient removal. Denitrifying bioreactors or wetland cells can be placed where shallow groundwater converges, while isotopic tools help verify removal pathways and detect bypass flow (29, 44, 59). In paddy systems, alternate wetting and drying should be paired with controlled flushing to avoid concentrating salts and nitrate (57, 60). Overall, by seeing farm fields as part of a connected biogeochemical landscape, CZA provides a framework for managing nutrients in relation to water flow paths.

### Soil salinization and land restoration

Irrigated agroecosystems worldwide face salinization from evaporation and inadequate drainage. CZA approaches rehabilitation by jointly addressing soils, shallow groundwater, and basin setting. Core measures include lowering water tables, leaching salts below the root zone when appropriate, amending sodic soils with gypsum, and using salt-tolerant or deep-rooted vegetation to protect structure and draw on shallow saline groundwater (39, 48, 49). A CZ-informed reclamation plan begins with three-dimensional mapping of salinity in soils and groundwater so that intervention can be targeted rather than uniform.

In China and other East Asian countries, several recent initiatives exemplify how integrating biophysical and human factors (“deep coupling”) and translating science into practice (“deep practice”) can foster more sustainable water use. For instance, High-Standard Farmland Construction (HSFC) in China modernizes fields through leveling, irrigation and drainage upgrades, soil improvement, and better access infrastructure. In a CZA frame, it is deep practice informed by deep depth (addressing subsoil constraints) and deep time (ensuring long-term productivity), implemented through engineered and managerial upgrades such as laser-leveled plots, lined canals or low-pressure pipelines, efficient pumps, storage ponds, and organic-matter enrichment (39, 48). Program evaluations and policy syntheses consistently report lower irrigation water use, reduced agrochemical inputs, and improved yield stability after HSFC implementation, even though exact magnitudes vary among regions. HSFC operationalizes farm-as-system thinking by jointly addressing shallow and deep flows, enabling conjunctive use, and building future adaptability. The model illustrates how CZA-type integration can be implemented at scale.

### Drought resilience and subsurface processes

Drought resilience depends partly on subsurface architecture. Weathered bedrock, deep regolith, shallow groundwater, and restrictive layers all influence how much water plants can access during prolonged dry periods (23, 24). CZA therefore promotes management that both improves access to subsurface stores and protects them: deeper rooting, alleviation of hardpans or acidity, conjunctive use of surface and groundwater, and managed aquifer recharge on suitable land (19, 27, 56). The key question is not only whether recharge increases storage, but whether it improves long-term quantity and quality under realistic operating conditions (54).

Field observations further indicate that plant roots can access rock moisture or perched water in fractures, and these hidden stores often determine whether trees survive severe droughts (23, 24). Management can leverage this by promoting deeper rooting, breaking restrictive layers, and timing irrigation to encourage depth exploration. In orchards, isotope and sap-flow measurements document seasonal shifts in water-source depth, supporting dynamic irrigation thresholds (61). At subfield scale, variable-rate irrigation that accounts for shallow groundwater and soil texture can stabilize yields and avoid overwatering wetter patches (50). More importantly, by treating the subsurface not as a static boundary but as part of the available water resource, farmers may improve drought preparedness by better accounting for subsurface water access.

### Cross-scale sustainability and sector integration

Farm-scale efficiency must align with basin-scale sustainability. Individually efficient farms can still deplete aquifers when withdrawals exceed recharge, so CZA explicitly links local actions to regional outcomes through basin dashboards, recharge and discharge mapping, groundwater monitoring, and policy instruments such as pricing, trading, pumping rules, or payments for recharge (18, 21, 37). It also highlights teleconnections through virtual water trade and urban-agricultural linkages such as wastewater reuse, both of which extend the consequences of local water decisions beyond the farm.

In practice, basin management plans informed by CZ-based recharge mapping and groundwater modeling can set withdrawal limits tied to long-term recharge and ecological baseflow needs (18, 21). Practical instruments include seasonal pumping caps, drought-stage triggers, and incentives for agricultural-MAR on suitable soils and geomorphic settings (56). CZA complements integrated water resources management (IWRM) by giving agriculture a measurable role in recharge, flood mitigation, and environmental flows. Transparent monitoring and open dashboards can help build the trust needed for performance-based plans and help prevent one sector's water savings from becoming another sector's loss.

Across these applications, CZA helps anticipate trade-offs early and align high-tech and nature-based measures with durable resilience rather than short-lived gains.

## Implications

Scientifically, CZA implies that agricultural water research should move from parallel disciplinary studies toward problem-focused integration. Adopting CZA reshapes science, management, and policy by treating farms as managed CZs with deep stores, long memories, and human coupling. It broadens agrohydrology's scope, challenges legacy practices, and catalyzes collaboration and innovation to better align agriculture with sustainable development goals.

### Advancing interdisciplinary science

CZA calls for integrated teams and long-term studies that jointly track weather, soils, roots, microbes, and management, while encouraging hybrid methods tailored to agroecosystems (20). Such collaboration can generate shared datasets, indicators, and decision tools that translate Critical Zone observations into practical guidance for farm- and basin-scale water management.

### Improving resource management and policies

CZA provides practitioners with a framework that explicitly links soil health to water security. For instance, water agencies can incorporate soil metrics (like infiltration capacity or organic matter levels) and groundwater recharge targets into regional water plans, acknowledging that a farm field's ability to absorb water is itself a form of water infrastructure (18, 41). This shifts agricultural water governance from practice-based compliance toward performance-based stewardship grounded in verified recharge, reduced leaching, and improved return-flow quality. Agricultural extension services can train farmers to interpret sensor data and model forecasts, making science-based management more accessible. Policy can incentivize practices with joint benefits—cover crops that raise soil carbon and also improve water retention, for example—thus integrating soil and water goals into one package (37).

### Bridging scales of governance

CZA connects local farm actions with watershed and global outcomes. Local water user associations or conservation districts could apply CZA models to align farm plans with basin targets, while national and global initiatives for climate, biodiversity, and water security can embrace agriculture as part of the solution, not just a problem to blame. For example, a country's climate plan might include soil carbon sequestration on farms as a water resilience measure. CZA's multiscale metrics help make these linkages concrete: farm-level indicators (like crop per drop, leaching risk, or soil carbon change) can roll up into basin dashboards showing aquifer trends and environmental flows (18). Those, in turn, inform national water security assessments and can even feed into international trade discussions via virtual water analyses (7). On the city–country interface, the framework helps municipalities partner with agricultural districts on source-water protection, wastewater reuse, and joint infrastructure for recharge or flood control (21).

### Enhanced resilience and sustainability

Embedding the four deeps through the 5M cycle equips landscapes with multiple buffers and feedbacks. Farms with healthy soils, strategic groundwater use, and integrated nature-based solutions (NBSs) are better able to withstand droughts and deluges, reducing crop losses, lowering runoff and erosion, and lessening downstream flood peaks. This may contribute to regional stability: aquifers are less likely to crash, and ecosystems receive more consistent baseflows and nutrient filtering, even under climate extremes. Essentially, CZA-managed systems have multiple buffers and feedback controls, which is the hallmark of resilience.

### Strengthening food and water security

By raising “crop per drop” while rebuilding soil and stabilizing groundwater, CZA strengthens national food security under climate stress. It reduces the risk of water-related crises. Regions that implement these practices may be less prone to water shortages that trigger food price spikes, migration, or tension between communities. In the bigger picture, CZA could help ensure that intensification of agriculture does not come at the expense of long-term viability of water resources, thus supporting global food security in a sustainable way.

### Overcoming institutional silos

Broader adoption requires coordination across agricultural, water, environmental, and planning agencies that often operate separately. CZA naturally bridges these domains, potentially spurring interagency working groups or joint soil–water boards. In academia and professional training, the goal need not be to replace disciplinary depth with a new generic degree. A more realistic priority is cross-training and shared literacy, producing scientists and practitioners who can communicate across agronomy, hydrology, geoscience, and policy while still retaining disciplinary expertise. That form of collaboration is more feasible and more useful than assuming that entirely new programs will solve the problem. Future training should not aim to produce superficially broad experts, but rather professionals with strong disciplinary foundations who can work effectively across shared data systems, indicators, and decision platforms.

### Public engagement and perception

Communicating CZA helps frame farms as stewards of aquifers, flood buffering, and biodiversity, building support for regenerative, water-aware practices and the policies that sustain them. Clear examples and locally relevant metrics can further help farmers, water agencies, and the public see how field-level actions translate into shared watershed benefits.

### Driving technological and economic innovation

Demand will grow for advanced tools like deep soil-moisture sensors, low-cost geophysical survey kits, open-source farm analytics, and crop varieties that exploit deeper soils or tolerate salinity. Entrepreneurs and researchers can innovate around CZA needs, e.g. developing simple recharge measurement tools or integrative advisory software. Economically, CZA reframes sustainability not as a cost but as risk management and value creation: fewer input losses (water and fertilizer), more stable yields in bad years, and even new revenue from ecosystem services or carbon credits. This may help attract investment and financing for farmers to adopt innovative practices, since there is a clearer return on investment.

### Limitations and caution

CZA is not a silver bullet; it must remain context-specific, data-informed, and adaptive. Monitoring capacity and model uncertainty vary widely, so implementation should begin with pilots, transparent assumptions, and adaptive management (35). Long-term legitimacy will also depend on co-production with farmers and attention to equity, especially for smallholders (36).

Overall, CZA shifts agriculture from short-term plot optimization toward long-term, basin-aware stewardship. By linking deep time, deep depth, coupled models, and deep practice, it offers a workable pathway to food and water security in a warmer, more variable world (18, 19, 21, 41).

## Future directions: toward resilient and smart agriculture

Agriculture is changing rapidly through new technologies, new contaminants, and new production systems. Whether the innovation is recycled wastewater, agrivoltaics, controlled-environment agriculture, or gene-edited rooting traits, the central question is the same: does it strengthen resilience across the managed CZ, or does it introduce new hidden liabilities? CZA provides a practical test for answering that question.

### Agriculture 4.0 meets CZA

Digital technologies—Internet of Things (IoT), artificial intelligence (AI), robotics, and data analytics—can strengthen every step of the 5M cycle when they are tied to hydrologically meaningful decisions rather than novelty alone. Multidepth sensors, weather stations, crop imaging, and smart valves can support selective irrigation, fertigation, and compaction remediation, while machine learning can help identify where additional measurement or model refinement is most needed (62). The opportunity is to move from prediction alone toward geographic decision intelligence that links observations to accountable intervention.

### Roadmaps by context

CZA implementation will vary across landscapes. Irrigated districts require a sequence of measured deliveries and pumping records, maps of recharge potential and hardpans, coupled crop-vadose-groundwater models, and pilot conjunctive-use schedules plus managed aquifer recharge tied to observed aquifer trends (18, 56). High-tech regions need field instrumentation, farmer-facing dashboards, district digital twins, and AI-guided variable-rate irrigation and fertigation, while also tracking slow soil-health indicators such as soil organic carbon and bulk density (45, 46). Saline–alkali landscapes require repeat EMI mapping, shallow-well monitoring, improved drainage, controlled leaching, gypsum or other amendments, and measures to prevent resalinization through water-table control and careful fertigation (48, 49). The common point is not that every landscape needs the same toolset but that each needs a context-specific pathway connecting measurement, diagnosis, intervention, and governance. These pathways should be treated as context-specific pilot sequences rather than fixed templates, because the same intervention may have very different hydrologic consequences across irrigated districts, high-tech farming regions, and saline–alkali landscapes. The practical objective is not to implement the full framework everywhere at once but to sequence interventions so that each step generates information that improves the next.

### Nature-based solutions as infrastructure

Buffers, grassed waterways, farm ponds, wetlands, and agroforestry regulate flow, trap sediment and nutrients, and add storage that stabilizes baseflow. CZA's contribution is to provide a canopy-to-bedrock logic for locating, evaluating, and governing these features across scales (26, 58).

### Smart, selective interventions

AI-guided variable-rate irrigation and fertigation, robotic subsoiling limited to compacted strips, and alerts derived from canopy temperature, soil-moisture profiles, and subtle subsidence can all support more selective action (45, 46). The key is to design these tools around farmer usability and decision relevance rather than technical novelty alone. Within CZA, AI is likely to be most useful when it extends beyond prediction toward geographic decision intelligence that translates heterogeneous observations into adaptive water-management actions.

### Digital twins and data assimilation

Future progress in CZA will depend heavily on better integration of observations, models, and decisions. Coupled crop-vadose-groundwater models can now assimilate satellite evapotranspiration and soil moisture, proximal geophysics, lysimeters, and hydrographs to build decision-ready digital twins (13, 29, 33, 51–53). Tagarakis et al. (63) confirm that digital twins are emerging as a practical interface between sensing, simulation, and real-time management across agricultural systems, although hydrologically explicit implementations remain limited. Ahsen et al. (64) further show, through a systematic review focused specifically on agricultural water management, that digital twins are moving from proof-of-concept toward operational relevance, especially when linked to AI-enabled scenario exploration. Used well, these systems support a test-implement-monitor-recalibrate workflow that helps identify robust options, expose unintended consequences, and detect thresholds before they become crises (35).

### Coupled decisions and shared dashboards

Farmer tools should make the hydrologic consequences of routine actions visible, while district dashboards coordinate deliveries, pumping, and environmental flows with transparent metrics and financing arrangements (21). A useful near-term strategy is to build minimum viable CZA pilots: a clearly defined problem, the smallest observation set needed to constrain it, a model or accounting framework to test interventions, and explicit success metrics.

### Deep depth—subsurface banking

Treat the soil–regolith–bedrock–aquifer continuum as a coupled store-and-supply system. Mapping infiltration “sweet spots,” verifying mounding, and pairing MAR with conjunctive-use rules can help stabilize aquifers across wet and dry years, but feasibility must be judged together with return-flow recovery, water rights, and climate constraints (23, 27, 55, 56).

### Deep time: climate readiness

Investment in slow variables such as soil organic carbon, stable structure, and perennial biomass can improve water holding, infiltration, and stress-year yields. Practical levers include deep-root phases in rotation, residue retention, controlled traffic, reduced tillage, and cover crops, all tracked with long-horizon indicators and trigger points (11, 12, 16, 40, 41).

### Operational priorities

Run the 5M cycle repeatedly at the management-unit scale. A minimum viable implementation can begin with a compact metric set—crop per drop, recharge rate, leaching risk, and shallow water-table trend—and link reporting, incentives, or compliance to those metrics. Success should be judged by measurable hydrologic outcomes under sustainability constraints, not by the sophistication of the technology alone.

In sum, the future under CZA is a learning agricultural system: farms and districts instrumented from canopy to aquifer, digital twins used to anticipate risk, landscapes designed with both engineered and nature-based storage, and institutions that reward hydrologic stewardship.

## Conclusion

Meeting future food demand under water scarcity and climate change requires agricultural water management that is deeper, more integrated, and more adaptive. CZA offers that shift by extending agrohydrology with deep time, deep depth, deep coupling, and deep practice, and by operationalizing those principles through the 5M cycle. Its practical value lies in linking better diagnosis to better decisions so that farms can sustain productivity while also supporting aquifer stability, water quality, carbon storage, and biodiversity. With interdisciplinary collaboration, shared data, and user-centered tools, CZA can help turn agriculture from a chronic stressor on soil and water into a contributor to long-term resilience in the Anthropocene.
